# Two-year Outcomes from a Single Surgeon's Learning Curve Experience of Oblique Lateral Interbody Fusion without Intraoperative Neuromonitoring

**DOI:** 10.7759/cureus.1980

**Published:** 2017-12-22

**Authors:** Kamal Woods, Ahtziri Fonseca, Larry E Miller

**Affiliations:** 1 Kettering Neuroscience Institute, Kettering Health Network; 2 Stanford Center for Clinical Research, Stanford University School of Medicine; 3 None, Miller Scientific Consulting

**Keywords:** fusion, lateral, lumbar, oblique, olif

## Abstract

Introduction

Oblique lumbar interbody fusion (OLIF) is a newer procedure that avoids the psoas and lumbosacral plexus due to its oblique trajectory into the retroperitoneal space. While early experience with OLIF is reassuring, the longer-term clinical efficacy has not been well established. The purpose of this study was to describe two-year clinical outcomes with OLIF performed by a single surgeon during the learning curve without the aid of the neuromonitoring.

Materials and methods

Chart review was performed for the consecutive patients who underwent OLIF by a single surgeon. Back pain severity on a visual analog scale (VAS) and Oswestry Disability Index (ODI) were collected preoperatively and postoperatively at six weeks, three months, six months, one year and two years.

Results

A total of 21 patients (38 levels) were included in this study. The indications for surgery were degenerative disc disease (n=10, 47.6%), spondylolisthesis (n=9, 42.9%) and spinal stenosis (n=6, 28.6%). The median operating room time was 351 minutes (interquartile range (IQR): 279-406 minutes), blood loss was 40 ml (IQR: 30-150 ml), and hospital stay was 2.0 days (IQR: 1.0-3.5 days). The complication rate was 9.5%, both venous injuries. There were no other perioperative complications. Back pain severity decreased by 70%, on average, over two years (p <0.001). A total of 17 (81%) patients reported at least a two-point decrease from the baseline. The ODI scores decreased by 55%, on average, over two years (p <0.001), with 16 (76%) patients reporting at least a 15-point decrease from the baseline. Over two years, no symptomatic pseudarthrosis, hardware failure, reoperations, or additional complications were reported.

Conclusions

The oblique lateral interbody fusion performed without the intraoperative neuromonitoring was safe and clinically efficacious for up to two years. The complication rate in this cohort is similar to other published OLIF series and appears acceptable when compared to the lateral lumbar interbody fusion (LLIF) and the anterior lumbar interbody fusion (ALIF). No motor or sensory deficits were observed in this study, supporting the premise that the neuromonitoring is unnecessary in OLIF.

## Introduction

Chronic non-specific low back pain presents a diagnostic and therapeutic dilemma to the clinician since the patient prognosis is poor when symptoms persist for six months or more [1-4]. In these patients, the lumbar interbody fusion is a surgical option that removes pain-generating compressive tissue, eliminates painful segmental motion, and restores sagittal balance [5]. The traditional surgical access corridors for lumbar interbody fusion include the anterior lumbar interbody fusion (ALIF), the lateral lumbar interbody fusion (LLIF), the transforaminal lumbar interbody fusion (TLIF), and the posterior lumbar interbody fusion (PLIF). Each approach has a distinct benefit-to-risk profile that is influenced by the anatomic structures that must be traversed to gain access to the interspace. Approaches such as ALIF and LLIF are associated with excellent clinical outcomes since access is straightforward and larger-sized implants can be used. However, the anterior approaches may be complicated by iatrogenic great vessel injury and anatomic restraints such as obesity and encumbrance of the iliac crest for accessing the Lumbar Spine (L4-5). Further, the neural injury with transient or permanent motor deficit is a major risk of LLIF. The posterior approaches largely avoid these risks but introduce a different set of challenges. The surgical corridor used with posterior PLIF and TLIF necessitates the muscle detachment, nerve root retraction and manipulation of the dura, with further iatrogenic injury risk related to impacted insertion of an interbody cage, especially in the presence of spondylolisthesis, and disc collapse [6-7].

Given these challenges, the minimally invasive retroperitoneal approaches to the anterior lumbar spine have gained popularity [8-9]. Until recently, these approaches have utilized a transpsoas trajectory which heavily is depended on neurophysiologic monitoring to avoid the neural injury. Due to the inability to directly visualize the psoas, the potential exists for transient motor and sensory disturbances [10-11]. Other drawbacks of these approaches include the inability to access lumbar vertebrae and vertebrae of the sacrum (L5-S1), challenges accessing L4-5 due to obstruction by the iliac crest, and difficulty identifying a safe path away from the lumbosacral plexus [12].

The oblique lateral interbody fusion (OLIF) has emerged as a minimally invasive procedure for degenerative lumbar diseases intended to mitigate challenges experienced with trans-psoas approaches. The goal of the oblique trajectory is to access the interspace while avoiding disruption of the psoas and lumbosacral plexus. The initial publications on OLIF have been encouraging, but have mainly focused on the technical aspects of the procedure, complications, and early results [13-16]. Yet, the scientific literature remains devoid of the long-term clinical outcomes with this technique. The purpose of this study was to describe two-year clinical outcomes with OLIF performed by a single surgeon during the learning curve without the aid of the neuromonitoring.

## Materials and methods

Study design

This was a retrospective study of the consecutive patients treated with OLIF by a single surgeon (Dr. Kamal Woods). This research received an exempt determination from the Western Institutional Review Board (Puyallup, Washington) and informed consent requirements were waived.

Patient selection

Each patient underwent a focused history and the physical examination. All the patients had failed prior conservative treatment, including the physical therapy and/or epidural steroid injection. The primary indications for the surgery were symptomatic degenerative disc disease, low-grade spondylolisthesis, or spinal stenosis. The primary diagnosis was confirmed by the magnetic resonance imaging and four-view X-rays.

Procedure

The OLIF procedure has been described in detail elsewhere [16]. Briefly, for fusion between L2-L5, the patient is positioned right lateral decubitus and secured on a radiolucent table with hips extended. The disc space of interest is marked on this skin and the vertical, lateral abdominal incision is made two fingerbreadths anterior to the disc space. For multilevel cases, the incision is centered between the surgical levels. The blunt finger dissection is performed through the lateral abdominal musculature into the retroperitoneum. Further dissection in the retroperitoneum is performed with direct visualization as the peritoneum is mobilized anteriorly. The disc space is identified anterior to the psoas muscle where annulotomy is made. During discectomy, trialing and interbody device placement, the surgical instruments initially enter obliquely, then are rotated vertically (orthogonal maneuver). For L5-S1 fusion, the disc space in approached between the bifurcation of the common iliac vessels. Sometimes, the left common iliac vein is mobilized to the left to achieve optimal exposure. The interbody device at L5-S1 was secured with a single buttress screw.

The patients were treated in a homogeneous manner in which polyetheretherketone PEEK interbody devices (Invibio Biomaterial Solutions, West Conshohocken, Pennsylvania) were used at L2-L5 (Medtronic Clydesdale, Memphis, Tennessee) and L5-S1 (Medtronic Sovereign). The interbody devices were packed with morselized allograft (Synthes demineralized bone matrix (DBX) mix). All the patients were supplemented with the posterior cortical screws at L2-L5 and sacral alar screws at S1 immediately after the OLIF. No bone morphogenetic protein was used in any case. No patient underwent posterior decompression. The neuromonitoring was not used during the lateral portion of the procedure.

Outcomes and follow-up

All patients in this series were followed by two years. The fusion status was assessed at each follow-up visit, with the anteroposterior and lateral x-rays at six weeks and three months and with flexion-extension x-rays at subsequent visits. In one case, pseudarthrosis was suspected and the computed tomography was performed. The perioperative data included the operative blood loss, operating room (OR) time (wheels into wheels out), hospital stay, and the complications. The potential complications included in the data review were vascular injury, blood transfusion, prolonged postoperative ileus (greater than three days), retrograde ejaculation, ureteral injury, renal injury, bowel injury, symptomatic pseudarthrosis, hardware failure, postoperative infection, incisional hernia, pseudohernia, reoperation, the neurological deficits (weakness, numbness, paresthesia), hip flexion pain, cerebrovascular accident, pneumonia, myocardial infarction, pulmonary embolism, deep vein thrombosis and sympathectomy affecting the lower extremities. The clinical and radiographic outcomes were collected during regularly scheduled office visits at pre-treatment, six weeks, three months, six months, one year, and two years. Back pain severity was assessed with a zero to 10 visual analog scale (VAS). The back function was assessed with the Oswestry Disability Index (ODI) version 2.0 [17], which is scored on a zero to 100 scale where lower scores indicate better back function.

Data analysis

The continuous data were reported as a mean and standard deviation or median and interquartile range (IQR), depending on normality assumptions. The categorical data were reported as frequencies and percentages. In patients who returned for follow-up but did not complete the VAS or ODI questionnaires, the missing values were imputed using the multiple linear regression with random error terms. The longitudinal changes in VAS and ODI were evaluated with repeated measures analysis of variance where statistical significance was set at p <0.05. The minimum clinically important difference (MCID) was defined as at least a two-point decrease in the VAS back pain scores [18-19] or at least a 15-point decrease in the ODI scores [18,20], respectively. The exploratory analysis of learning curve effects was also performed. The outcomes by learning curve period were compared with a Wilcoxon-Mann-Whitney test or Fisher’s exact test. Given the potential for patient characteristics to vary across the learning curve, comparisons were adjusted to account for the baseline patient characteristics using propensity-score covariate adjustment that included the sex, age, body mass index, number of levels, and L5-S1 involvement. The data were analyzed with Statistical Analysis System SAS v.9.4 (SAS Institute, Cary, North Carolina).

## Results

From October 2013 to February 2015, 21 consecutive patients were treated with OLIF by a single surgeon. These cases represent the first 21 OLIF procedures performed by the primary author (KW). The baseline patient characteristics are shown in Table 1. The mean age was 62 years and 67% of the patients were male. The primary surgical indications were degenerative disc disease (48%), grade I spondylolisthesis (43%), and spinal stenosis (29%). The patients were treated at one level (48%), two levels (24%), or three levels (29%). Six (29%) patients underwent fusion at L5-S1. The median OR time was 351 minutes (IQR: 279-406 minutes), the blood loss during the entire anterior and posterior procedure was 40 ml (IQR: 30-150 ml), and the hospital stay was 2.0 days (IQR: 1.0-3.5 days) (Table 2).

**Table 1:** The baseline patient characteristics. The values are mean±standard deviation or count (percentage) unless noted otherwise. The patients may present with multiple surgical indications.

Characteristic	Value
Demographics	
Male sex	14 (67)
Age, yrs	62±11
Body mass index, kg/m^2^	27±5
Medical history	
Tobacco history	11 (52)
Obesity	4 (19)
Hypertension	3 (14)
Clinical status	
Back pain severity	6.1±2.0
Oswestry Disability Index	51±14
Indication for surgery	
Degenerative disc disease	10 (48)
Spondylolisthesis, grade I	9 (43)
Spinal stenosis	6 (29)
Spondylosis	2 (10)
Radiculopathy	2 (10)
Lumbar hypolordosis	1 (5)

**Table 2:** The periprocedural outcomes. The values are median (interquartile range) or count (percentage). The operating room time includes the oblique lateral interbody fusion and posterior fixation. L: Lumbar vertebrae, S: vertebrae of the sacrum.

Characteristic	Value
Blood loss, ml	40 (30-150)
Operating room time, min †	351 (279-406)
No. treated levels	
1	10 (48)
2	5 (24)
3	6 (29)
Treated level	
L2-L3	5 (24)
L3-L4	9 (43)
L4-L5	18 (86)
L5-S1	6 (29)
Hospital stay, days	2.0 (1.0-3.5)

Two intraoperative venous injuries occurred. In the second case of this series, a left iliac vein was injured at L5-S1 and required intraoperative repair. The patient lost 1200 ml blood but did not require transfusion. In the nineteenth case of the series, bleeding from the iliolumbar vein was observed, and was controlled with Gelfoam® and thrombin. There were no other perioperative complications. Notably, the neurophysiologic monitoring such as electromyography (EMG), somatosensory evoked potentials (SSEP), and motor evoked potentials (MEP) was not used in this study and there were no cases of the neurological injury. The overall complication rate was 9.5%. When adjusting for the patient characteristics, there were no differences in the blood loss, OR time, or perioperative complications during the learning curve. However, the median length of hospitalization decreased significantly during this period, from 3.5 days to 1.0 days (Table 3).

**Table 3:** The comparison of periprocedural outcomes during the learning curve. The values are median (interquartile range) or count (percentage). The operating room time includes the oblique lateral interbody fusion and the posterior fixation. The propensity-score covariate-adjusted using sex, age, body mass index, number of levels, and L5-S1 involvement.

Characteristic	First 10 cases	Last 11 cases	P-value
Blood loss, ml	100 (30-188)	30 (20-100)	0.41
Operating room time, min	397 (289-443)	347 (271-393)	0.33
Hospital stay, days	3.5 (1.0-4.3)	1.0 (1.0-2.0)	0.03
Complication	1 (10)	1 (9)	0.70

**Figure 1:**
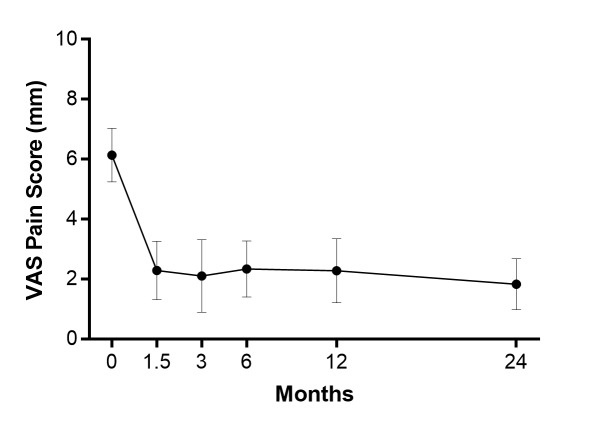
Back pain severity over two years following the oblique lateral interbody fusion. Back pain severity at two years was statistically lower relative to preoperatively (p <0.001). The values were mean and 95% confidence interval. VAS: visual analogue scale.

**Figure 2:**
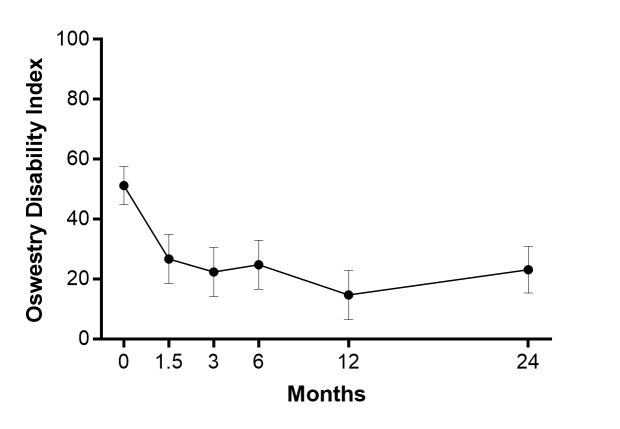
Oswestry Disability Index (ODI) over two years following the oblique lateral interbody fusion. The ODI at two years was statistically lower relative to preoperatively (p <0.001). The values were mean and 95% confidence interval.

All patients returned for a two-year follow-up. The back pain severity decreased by 70%, on average, over two years (p <0.001) (Figure 1). A total of 17 (81%) patients achieved the MCID threshold of two-point decrease for back pain severity. The ODI scores decreased by 55%, on average, over two years (p <0.001) (Figure 2). A total of 16 (76%) patients achieved the MCID threshold of 15 point decrease for the ODI. Over two years follow-up, no symptomatic pseudarthrosis, hardware failure, or additional complications were detected. No patient required re-operation at any lumbar level during follow-up.

## Discussion

The increasing adoption of minimally invasive techniques for the spine surgery in recent years has led to significant advancements in instrumentation for lumbar interbody fusion. The OLIF technique allows an oblique-lateral trajectory to access multiple lumbar levels without the patient repositioning while preserving the psoas muscle. This retrospective study demonstrates that OLIF can be safely performed during the learning curve with favorable two-year outcomes. There were several unique aspects of this study, including the longest term OLIF results available, all cases being performed during the learning curve, and without the use of the neuromonitoring. Since that time, the procedure has been further refined, including the development of retractors and instruments specifically suited to the oblique trajectory, which may potentially further improve the safety outcomes.

Previously published OLIF series [16] reported a postoperative ileus rate of 2.9%. That rate likely reflects in part of the challenges seen with the initial retractors that were used for OLIF. As refinements in the procedure have taken place, the rate of ileus has decreased. No cases of postoperative ileus were seen in this cohort. Overall, our results compare favorably to those reported in a systematic review of OLIF outcomes [21]. These authors reported a mean blood loss of 110 ml, six-day hospital stay, and overall complication rate of 11%. In comparison, the results from the current series were 40 ml blood loss, two-day hospital stay, and 10% complications. Further, it has been shown that operator experience does not influence the rate of complications with OLIF [15]. We observed the same finding although low complication rates and small sample size limited our ability to detect such trends. We also noted that even when controlling for the patient characteristics, the hospital stay duration decreased significantly over the learning curve. This was largely due to the conservative patient management attributable to the utilization of a new technique with the early cases, followed by increasing knowledge of the expected postoperative course with few complications allowing faster discharge.

The favorable safety profile of the OLIF procedure is largely attributable to the trajectory that precludes the need for the neuromonitoring. The OLIF allows the direct visualization of the psoas, anterior longitudinal ligament, and disc space. Further, since the neuromonitoring is not required, greater relaxation of the abdominal wall muscles can be achieved which may also allow for a smaller incision. This also allows for the psoas to be more easily retracted to increase the area of the oblique corridor at L2-L5 between the abdominal aorta and psoas.

Through the patient evaluation and selection including careful attention to proper surgical techniques are the key factors to achieve satisfactory outcomes with OLIF. The patients with active infection, metabolic bone disease, severe osteoporosis, malignancy, local fracture, grade II/III spondylolisthesis, morbid obesity, or degenerative disc disease affecting four or more levels add complexity to the OLIF. Also, attention should be paid to the potential for anatomic variants, especially of the lower lumbar segmental arteries [22], which could result in the iatrogenic injury. 

The LLIF is often challenging at the L4-L5 level. The factors that contribute to this challenge are high iliac crest that hinders access to the L4-L5 disc space, and more anteriorly-located psoas muscle and femoral nerve limiting the safe working window. By using a more anterior incision and an oblique trajectory, the OLIF avoids the iliac crest and femoral nerve. No jackknifing is necessary for OLIF and this may decrease the risk of femoral injury. No nerve injury was seen in this series and in larger OLIF series [16].

The novelty of this study is that the OLIF remains safe and effective through two years follow-up despite all the cases performed during a surgeon’s learning curve and without the neuromonitoring. A key advantage of OLIF is the ability to access all the lumbosacral levels in one position, including L5-S1. This study addressed the learning curve associated with this strategy of one-position surgery. There were also several limitations of this study that warrant mention. This was a retrospective study of only 21 patients treated by a single surgeon. Therefore, the potential for bias, concerns regarding the generalizability of findings, and limited ability to detect rare complications must be acknowledged. Also, all the patients underwent OLIF with no control group; therefore, comparative safety and the effectiveness related to other interbody fusion approaches cannot be determined by this research. Finally, the computed tomography (CT) imaging during the follow-up was reserved only for one patient with suspected pseudarthrosis based on the clinical symptoms and X-ray findings. Thus, this study has limited ability to detect asymptomatic pseudarthrosis.

## Conclusions

Oblique lateral interbody fusion performed without the intraoperative neuromonitoring was safe and clinically efficacious for up to two years. The complication rate in this cohort is similar to other published OLIF series and appears acceptable when compared to LLIF and ALIF. No motor or sensory deficits were observed in this study, supporting the premise that the neuromonitoring is unnecessary in the OLIF.
